# Social Media and the Transformation of the Physician-Patient Relationship: Viewpoint

**DOI:** 10.2196/25230

**Published:** 2021-12-24

**Authors:** Ella M E Forgie, Hollis Lai, Bo Cao, Eleni Stroulia, Andrew J Greenshaw, Helly Goez

**Affiliations:** 1 Department of Anthropology University of Alberta Edmonton, AB Canada; 2 Department of Dentistry University of Alberta Edmonton, AB Canada; 3 Department of Computing Science University of Alberta Edmonton, AB Canada; 4 Department of Psychiatry University of Alberta Edmonton, AB Canada; 5 Department of Pediatrics University of Alberta Edmonton, AB Canada

**Keywords:** social media, social determinants of health, precision medicine, patient care

## Abstract

As many as 80% of internet users seek health information online. The social determinants of health (SDoH) are intimately related to who has access to the internet and health care as a whole. Those who face more barriers to care are more likely to benefit from accessing health information online, assuming the information they are retrieving is accurate. Virtual communities on social media platforms are beginning to serve as venues for seeking health information online because peers have been shown to influence health behavior more than almost anything else. As a positive mediator of health, social media can be used as a direct or indirect mode of communication between physicians and patients, a venue for health promotion and health information, and a community support network. However, false or misleading content, social contagion, confirmation bias, and security and privacy concerns must be mitigated to realize the full potential of social media as a positive mediator of health. This paper presents the shifting dynamics of how such communities are affecting physician-patient relationships. With the intersections between the SDoH, social media, and health evolving, physicians must take into consideration these factors when establishing their relationships with patients. We argue a paradigm shift in the physician-patient relationship is warranted, one where physicians acknowledge the impacts of the SDoH on information-seeking behavior, recognize the positive and negative roles of social media as a mediator of health through the lens of the SDoH, and use social media to catalyze positive changes in the physician-patient relationship. We discuss how the physician-patient relationship must evolve to accommodate for the ever-increasing role of social media in health and to best use social media as a tool to improve health outcomes. Finally, we present a fluid and multicomponent diagram that we believe will assist in framing future research in this area. We conclude that it is ineffective and even counterproductive for physicians to ignore the relationship between social media, the SDoH and health, their impact on one another, and the effect it has on designing the medical encounter and the delivery of care under the definition of precision medicine.

## Introduction

Precision medicine, a comprehensive approach to patient care that takes into consideration genetics and the social determinants of health (SDoH) when diagnosing and treating disease [1,2], is prevailing among the priority areas for research in Canada and should be considered the standard for patient care worldwide [3]. The SDoH include socioeconomic factors that affect health and well-being, such as income, education, and employment [4]. Such factors are affected by societal systems of oppression and are intimately linked to an individual’s position in society relative to the societal status quo. As neither genetics nor the SDoH alone can indicate health status, the two must be viewed as intrinsically and inextricably linked to one another and thus to an individual’s overall health and well-being [5]. By definition, a precision health care approach should be both personalized to the patient and humanistic. With a multitude of health factors that can influence and are influenced by the SDoH, they should be considered by physicians when managing their patients’ health concerns. However, many SDoH and barriers to care issues continue to be overlooked by physicians [6-8].

Research has shown that as many as 80% of internet users seek health information online [9,10]. Social media platforms, internet-based user-driven community platforms for communication and sharing user-created content [11], have flourished as virtual communities where people exchange information and opinions, and seek support and advice from peers [12-14]. Such platforms include blogs (eg, WordPress), wikis (eg, Wikipedia), social bookmarking (eg, Reddit), social network sites (eg, Facebook), status update services (eg, Twitter), virtual world content (eg, Minecraft), and media-sharing sites (eg, YouTube) [11]. Specifically, health information seeking is among the most popular online activities with diet/nutrition, physical activity, signs and symptoms, treatment, and public health interventions as some common examples [8-10,12,13,15]. Social media has been studied in attempts to improve health outcomes with mixed results, but it is not yet clear what aspects of successful interventions precipitated their success [16]. Likewise, the reasons for failure of unsuccessful interventions, which led to worsened health outcomes or increased health inequity, are not clear. Thus, social media has the potential to positively moderate patient health, but how it can be used correctly to maximize benefit and minimize repercussions for patients remains to be seen.

In part related to the accepted importance of understanding patients’ voices and perspectives, it has been implied that social media use should also be taken into consideration by physicians as a determinant of health [6]. Although health care practitioners remain the principal trusted authority for health information, the SDoH of the patient—including accessibility barriers such as geography, cost, and time—result in patient preference for online searching over in-person consultations [12]. Due to its social connectedness, social media is one of the preferred venues for obtaining health information and community support, and peers have been shown to influence health behaviors more than almost anything else [14]. There are questions as to the accuracy and unbiased nature of online health information, especially that which is disseminated on user-created content platforms such as social media [8,17,18]. Further, an individual’s internet competency does not necessarily equate to their medical literacy. However, whether or not social media is a net positive or negative mediator of health, as we will argue, it undoubtedly affects individual health status in unique and substantial ways and thus cannot be ignored by physicians.

In this paper, we examine the complex relationship between social media and the SDoH. We propose that social media’s effects on health should be considered as part of the standard of care. Moreover, we argue that a paradigm shift in the physician-patient relationship is warranted, one where physicians acknowledge the impacts of the SDoH on information-seeking behavior, recognize the positive and negative roles of social media as a mediator of health through the lens of the SDoH, and use social media to catalyze positive changes in the standard of care. We suggest that only by broadening our understanding of the intimate linkages between social media and the SDoH, and incorporating it as part of patient care, can the gap be bridged effectively to deliver a vision of precision medicine that is inherently and sufficiently personalized.

## The Impacts of the Social Determinants of Health on Information-Seeking Behavior

The Government of Canada recognizes 12 determinants of health, including but not limited to biology and genetic endowment, childhood experiences, physical environment, and access to health services [4]. Not all determinants of health are SDoH. Rather, the SDoH are those that focus on social and economic factors such as race, income and social status, education and literacy, and employment and working conditions. The World Health Organization’s Commission on the Social Determinants of Health has defined SDoH as “the conditions in which people are born, grow, live, work and age” [19].

It is imperative to consider who has access to the internet when discussing the demographics of social media users, as social media users are by definition a subset of internet users. Access to the internet in general, a requisite for accessing social media, is related to factors underlying the SDoH. This relationship between internet access and the SDoH influences what information individuals are accessing and how they are accessing it [20,21]. This, in turn, affects their use of digital tools such as social media, which encompasses a wide array of websites and applications. Nearly 60% of the global population have access to the internet [22]. Nevertheless, a digital divide exists, and although internet use increases every year, this digital divide highlights disparities in access for underserved populations, especially those in low- and middle-income countries (LMIC) [14,22,23].

Age, an SDoH, is also a factor contributing to the differentiated use of social media; older adults tend to search for health information online significantly less than younger age cohorts such as Generation Y (1977-1990) because they lack prior internet experience and thus possess lower internet competency [8,14,24,25]. In addition, evidence indicates that education, another recognized SDoH, and specifically higher education correlates with an increased likelihood of searching for health information online as does identifying as female [20,26].

Overall, people of low socioeconomic status or from LMIC, older adults, and less educated individuals face the most substantial barriers to accessing the internet in general and social media in particular. Unfortunately, this is the same population who stand to benefit the most, healthwise, from having access to health information online [14]. The apparent need for internet access in specific SDoH segments reinforces clearly the statement delivered by the United Nations Human Rights Council that access to the internet is a basic human right [27].

## Social Media’s Potential as a Positive Mediator of Health

Accessibility barriers to in-person care are currently unacceptably high [14,22,23], and methods of communication between physicians and patients remain strictly in traditional formats [7]. Despite enormous advancements in internet technology and virtual communication, patients are still required to appear in-person at a physician’s office to communicate with a physician and receive care. To do so requires substantial privilege on the part of the patient, such as having the time and resources to attend an appointment. Recently, due to the COVID-19 pandemic, there has been an acute increase in emphasis on providing more virtual care. However, this is still quite limited and “temporary” as “there isn’t a lot of readiness” for using virtual health care [28]. Although methods of communication between physicians and patients are “at the heart of healthcare” [7], they are traditionally designed using practices that appear to lag substantially behind modern methods, in part due to regulatory bodies’ regulations and practice guidelines [28]. Although patients tend to seek health information online for a number of reasons, the majority state that they would prefer to obtain this information from health care practitioners, but it is simply not within their means to do so due to an inability to access care as a result of economic, social, cultural, or physical barriers [23,29].

Social media is largely free, easy to access from multiple geographical locations, and considered by patients to be “more convenient, timely, cost-effective” [8]. In some cases, receiving care in person may be impeded by stigma (eg, for treatment of mental health or sexually transmitted infections). In that regard, social media is reported by users as “privacy protective, and [less embarrassing]” [8] as a venue for seeking health information. Such perceptions are not always accurate, as will be discussed in the following section, and they by no means represent all social media users [30]. Further, social media is inclusive; provides a sense of solidarity, hence enhancing the attribute of community support; and grants a greater perceived sense of control to patients over their own health [29].

Patients have reported that information received from physicians during in-person consultations was “not clear, satisfactory, or conductive for asking additional questions” [31]. This may be one of the reasons that patients turn to social media for health information. When this occurs, they report feeling more knowledgeable, confident, and empowered in their abilities to communicate with health care practitioners [6]. Patient satisfaction (the perceived standard of care) is thus improved when social media is used as a tool for obtaining health information.

Perhaps equally important to decreasing accessibility barriers and increasing patient access to health information, social media also acts as a support network [12,13]. One study found that support networks are a preferred venue for obtaining health information, second only to physicians [12]. Support networks, including those facilitated by social media, are linked to the SDoH [32-34] and substantially affect health behaviors [35]. This is useful for health promotion and health outcomes, as people may be persuaded to partake in positive health activities such as healthy diet, exercising [36-38], and receiving their annual flu shot if their peers have posted publicly about participating in these activities [39]. In sum, social media has the ability to mitigate the SDoH that result in limited physical accessibility, enhance personal confidence, and empower patients’ communication with their physicians.

## Social Media’s Potential as a Negative Mediator of Health

For all the aforementioned benefits, social media is not exclusively a positive mediator of health [29,40]. There are notable problems with obtaining health information on social media; direct implications of social media use on health; and a problematic, homogeneous, “single story” narrative that is presented. It is important to acknowledge the negative and potentially dangerous effects of social media on health to reconcile them.

Social media tends to present information of questionable credibility, and it is oftentimes sponsored by a potentially biased entity [8]. Occasionally, online health information is entirely false [17]. In addition, although it may provide patients with a sense of privacy in comparison to discussing stigmatized topics in person with physicians, there are notable concerns about anonymity and privacy when obtaining health information online [29]. Confirmation bias (ie, the tendency to seek and trust information that is belief consistent and to discredit information that is belief inconsistent) is another well-documented danger associated with seeking health information online [18]. Personalization algorithms on social media platforms can further polarize the information available to patients though “recommended” or “suggested” content [13,41]. This content is automatically sourced based on previous social media activity, and it is presented to the user whether they are seeking it or not. Antivaccination content is a particularly good example, as parents who search for vaccine information online are more likely to hold antivaccination beliefs and possibly be active on similar communities of interest [10,18].

Emerging literature is increasingly documenting the direct impacts of social media on health [13,35]. Social media has been found to promote a sedentary lifestyle, increase self-isolation, decrease quantity and quality of sleep, and negatively impact mental health [13]. Social contagion, a recently documented phenomenon, depicts the contagious nature of certain noncommunicable health conditions over social media, including obesity and emotions such as happiness, anxiety, and depression [35]. Social influence and peer recommendations may substantially alter a person’s health behaviors as well, likely due to susceptibility to peer pressure, desire to belong to a group or feel supported, and perception of credibility of the recommendations [14,35,42]. A vicious cycle between anxiety and online health information seeking is another documented phenomenon, where high levels of anxiety are associated with online health information seeking, the findings of which further increases anxiety [43].

In 2009, Chimamanda Ngozi Adichie delivered a now renowned TEDtalk about the dangers of a “single story” narrative. Single stories emerge when only one narrative about a group of people is widely shared and accepted. It then becomes assumed that all members of said group shared the same experience, one that aligns with the single story. Recently, this concept was extended to stories of health experiences shared on social media [40]. An anthropological study found that single stories of health experiences (surrogacy, in this particular example) are disseminated widely across social media platforms. Not only do these single stories ignore variation in individual experiences and personal SDoH, but they also tend to hold little truth at all. Any deviation from the single story on social media, including any disclosure of personal experiences, is discredited and rejected, dehumanizing those who do so. Physicians must be made aware of the homogeneity of information on social media and how this may affect their patients’ perceptions of health and health experiences. Further, patients using social media as an online community wherein they may communicate with others with similar health conditions must be empowered to tell their own story while acknowledging that it may not align with the stories of others. Subjective recounts of disease are not necessarily misinforming so long as patients understand the difference between individual perception and experience of disease versus etiology and treatment of disease, the latter of which necessitates a degree of prerequisite medical knowledge that goes above and beyond digital literacy [44,45].

## Using Social Media to Catalyze Positive Changes in the Physician-Patient Relationship

Although the positive role of social media as a mediator of health is ample (ability to improve methods of direct and indirect communication between physicians and patients, increase access to health information, and foster a community support network), it is also imperative to address and mitigate the negative aspects of social media, such as security and privacy concerns. Work must be done on the part of the physician and the patient to shift the physician-patient relationship toward one that is inclusive of the role that social media plays in health and that uses social media as a tool to promote health and well-being.

During in-person consultations, physicians must be open to discussing the roles of social media as a mediator of health. In a recent interview, patients perceived health care workers to be in “overt or tacit opposition” [6] to any mention by patients of health information retrieved online. Such mentality reflects the traditional physician-patient relationship where patients hold little autonomy over their own medical journey [23]. Take, for example, a scenario wherein a patient, Cindy, a 24-year-old veterinary technician, discloses to her physician concerns around the COVID-19 vaccines. Cindy is a first-generation immigrant living in a multigenerational home and is worried that she may become contagious with the virus immediately after immunization, posing a health risk to her older adult immunocompromised grandmother. If her grandmother were to fall ill, Cindy fears that her family would not be able to afford the necessary care. Therefore, Cindy feels it is best for her family not to get the vaccine. For the physician to mitigate these concerns, it is imperative that they attempt to discern from where these anxieties originate. To write off Cindy’s beliefs simply because they are biomedically unfounded and derived from information sought on social media could be counterproductive in protecting or improving her health.

Further, social media may be used as an indirect line of communication between physicians and patients, if used as a venue for health knowledge dissemination and translation for the purpose of health promotion [46,47]. To elaborate, physicians or health care experts may communicate with their patients on social media via public posts about health-promoting behaviors they recommend and providing them with trusted links for further information. In addition, physicians may refer their patients to patient-driven online advocacy groups such as the Light Collective, a nonprofit organization that, among other objectives, works to shine a light on privacy breaches and so-called “bad data-sharing” [30]. This would aid in decreasing misinformation and diversifying the pervasive single-story narratives that encapsulate many health conditions. We recognize that it is not only up to the physician to identify and quell misinformation on social media; it is largely up to the platform itself to create regulatory policies and practices—or better yet, algorithms to identify problematic posts [48]—to minimize false or misleading content and to ensure that it is not being amplified in “recommended” or “suggested” content. In the previously discussed scenario, it is possible that Cindy would not have developed such concerns over the COVID-19 vaccines had she viewed credible biomedical data, or conversely, had she not viewed biomedically unfounded data, on social media. This is especially likely if Cindy is passively consuming data (ie, not actively seeking data).

A patient’s level of education and literacy as an SDoH impacts their level of medical literacy [49], which is only as good as the information they have acquired; it may be rendered obsolete if the knowledge they have gained is inaccurate. Therefore, patients should feel empowered to ask questions during their in-person consult or through anonymous or confidential online forums wherein knowledgeable health care practitioners provide answers. Although it is important for physicians to provide health care information to patients in terms that they are able to understand, we posit that it may also be beneficial to provide patients with a list of the formal medical terms associated with their condition. This may improve the caliber of search results they find online and reduce the likelihood of conflating different conditions or symptoms based on the colloquial descriptions given to them by their health care provider. Finally, patients should be encouraged to approach social media platforms in the context of health with curiosity and skepticism, embracing the community and solidarity aspects they may provide to those facing similar health problems while ensuring that objective medical data are not trusted until substantiated by scholarly sources or health care practitioners. For Cindy, a list of search terms will allow her to return home and actively seek reputable information on the COVID-19 vaccines. On the contrary, a lack of take-home information may result in Cindy searching what she knows—“contagious after COVID-19 vaccine”—which may elicit misguiding results that confirm her bias. A brief but thorough discussion with her physician about the dangers of passively consuming bad data on social media may also prevent such encounters in the future.

## Summary

**Figure 1:**
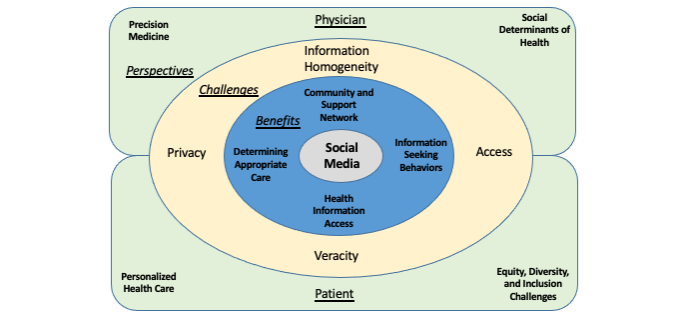
A fluid and multicomponent diagram to assist in framing future research around social media and the transformation of the physician-patient relationship.

In this paper, we reviewed the complex, multifaceted, and dynamic intersections of social media and SDoH in the era of precision medicine. We argued for its inclusion as part of routine patient care and demonstrated the potential of social media to be used as a positive mediator of health so long as its negative mediation effects are minimized. Further, we discussed how the physician-patient relationship must shift to accommodate for the ever-increasing role of social media in health and best use social media as a tool to improve health outcomes. These intricacies warrant further research. However, to conceptualize our study into a framework of understanding and development in this area, we present Figure 1 that we believe captures the potential challenges and areas of contention in bringing social media into precision medicine.

1. The benefits of social media use in health care are listed in the first ring surrounding social media. Social media can act as a support network, promoting information-seeking behaviors, allowing patients to determine their own appropriate care, and enabling health information access, all affected by and affecting the SDoH.
2. However, social media also brings challenges to promoting health, as is illustrated in the second ring. Although privacy and access to social media are common issues, the public also faces the issue of veracity of information. Further, patients face an information homogeneity problem in the form of social media’s single story, which does not account for diversity of experiences embodied by the SDoH.
3. Physicians and patients have different perceptions of how social media may be used in relation to health care, largely due to miscommunication. Although the public expects to receive personalized health care, physicians deliver their version of this as precision medicine. In addition, although the public perceives societal issues as equity, diversity, and inclusion challenges, physicians understand these issues as they relate to health and categorize them as the SDoH. This is not an issue of incongruity but one of language. Communication barriers must be acknowledged to be overcome.
4. Future research in this domain needs to recognize the complex dynamics of how social media interacts with the SDoH to develop solutions that can comprehensively improve the delivery of health care in the future.

## Conclusion

As the role of social media in health evolves, new directions of research are needed to better understand the impacts of social media on health and inform physicians on how it can be integrated as part of patient care. Our discussion posits that health and social media are intimately linked through the prism of the SDoH and that this linkage is only amplifying over time. We argue that it is thus ineffective and even counterproductive for physicians to ignore this relationship and the impact it has on designing the medical encounter and the delivery of care. For physicians to deliver the highest standard of care under the definition of precision medicine, the complex interaction between social media and the SDoH and their impact on one another must be taken into consideration.

## Abbreviations

LMIC: low- and middle-income countries
SDoH: social determinants of health
